# Obesity Induces Artery-Specific Alterations: Evaluation of Vascular Function and Inflammatory and Smooth Muscle Phenotypic Markers

**DOI:** 10.1155/2017/5038602

**Published:** 2017-03-30

**Authors:** Antonio Garcia Soares, Maria Helena Catelli de Carvalho, Eliana Akamine

**Affiliations:** Department of Pharmacology, Institute of Biomedical Sciences, University of São Paulo, São Paulo, SP, Brazil

## Abstract

Vascular alterations are expected to occur in obese individuals but the impact of obesity could be different depending on the artery type. We aimed to evaluate the obesity effects on the relaxing and contractile responses and inflammatory and smooth muscle (SM) phenotypic markers in two vascular beds. Obesity was induced in C57Bl/6 mice by 16-week high-fat diet and vascular reactivity, mRNA expression of inflammatory and SM phenotypic markers, and collagen deposition were evaluated in small mesenteric arteries (SMA) and thoracic aorta (TA). Endothelium-dependent relaxation in SMA and TA was not modified by obesity. In contrast, contraction induced by depolarization and contractile agonists was reduced in SMA, whereas only contraction induced by adrenergic agonist was reduced in TA of obese mice. Obesity increased the mRNA expression of pro- and anti-inflammatory cytokines in SMA and TA. The expression of genes necessary for maintaining contractile ability was increased by obesity, but the increase was more pronounced in TA. Collagen deposition was increased in SMA, but not in TA, of obese mice. Although the endothelial function was still preserved, the SM of the two artery types was impaired by obesity, but the impairment was higher in SMA, which could be associated with SM phenotypic changes.

## 1. Introduction

According to the World Health Organization in the last year about 39% of adults were considered obese [1]. Insulin resistance, dyslipidemia, hyperleptinemia, oxidative stress, low-grade systemic inflammation, and hypertension are consequences of a dietary behavior in which there is predominance in the amount of fat ingested [2–7].

Endothelial cells have a central role in the vascular homeostasis by releasing, among others, nitric oxide synthase- (NOS-) derived nitric oxide (NO), prostaglandins, angiotensin, and endothelin. Endothelial dysfunction is the most common vascular alteration observed in cardiovascular and metabolic diseases [3, 8, 9]. On the other hand, smooth muscle cells provide vessel wall structure and, by contracting and relaxing, are responsible for regulating vascular tone, maintaining the intravascular pressure and tissue perfusion. Smooth muscle cells acquire a phenotype that ranges from contractile/quiescent to a synthetic/proliferative phenotype [10]. In adult individuals, smooth muscle cells with contractile phenotype are predominant in the vascular wall, but in vascular repair/injury conditions differentiated smooth muscle cells switch to a synthetic phenotype [11, 12].

Structural and functional vascular alterations are expected to occur in obese individuals and the raise of proinflammatory factors due to obesity development plays an important role in the vascular dysfunction and cardiovascular disease [13–17]. Endothelial and smooth muscle cells present different characteristics depending on the artery type, since the embryogenic origin and function of large conduit and small resistance arteries are different [18–20]. Thus, the impact of obesity could be different depending on the artery type, but in general studies evaluate a single artery type.

The objective of this study was to evaluate the effects of high-fat diet-induced obesity for 16 weeks on the relaxing and contractile responses and inflammatory and smooth muscle phenotypic markers in two vascular beds, which have different functions.

## 2. Materials and Methods

### 2.1. Animals and Induction and Characterization of Obesity

All experimental procedures were performed in accordance with the Brazilian National Law and were approved by the by Ethics Committee of the Institute of Biomedical Sciences (ICB), University of São Paulo (USP) (number 195/11). Four-week-old C57BL/6 male mice were divided into two groups: (1) control group, which was fed a regular chow (70% carbohydrate, 20% protein, and 10% fat), with a caloric content of 3.8 kcal/g; (2) obese group, which was fed a high-fat diet (26% carbohydrate, 15% protein, and 59% fat) (PragSoluções, Brazil), with a caloric content of 5.4 kcal/g. The animals were maintained on a 12:12-hour light-dark cycle in a temperature-controlled environment (22 ± 2°C) with free access to food and tap water.

After 16 weeks subjected to a high-fat diet, the animals were fasted for 6 hours, weighed, and anesthetized with intraperitoneal injection of ketamine (150 mg/kg) and xylazine (7.5 mg/kg). Periepididymal and retroperitoneal fat deposits were excised and weighed. Blood samples were collected by cardiac puncture and the serum was used for biochemical assays. Triglycerides and total, HDL, and LDL cholesterol levels were measured by colorimetric kit assays (Labtest, Brazil). Glucose level was assessed by reactive strips (Johnson & Johnson, USA). Insulin (Cayman Chemical, USA) and TNF-*α* and IL-6 (BD Biosciences, USA) levels were assessed by ELISA assay kit.

### 2.2. Vascular Reactivity

After anesthesia, the mesenteric bed and the thoracic aorta were harvested and immediately placed in cooled Krebs-Henseleit solution (in mM: 130 NaCl, 4.7 KCl, 14.9 NaHCO_3_, 1.6 CaCl_2_·2H_2_O, 1.18 KH_2_PO_4_, 1.17 MgSO_4_·7H_2_O, 0.026 EDTA, and 5.5 glucose). After removing fat and connective tissue from both vascular beds, segments (2 mm in length) were transferred into wire myography chambers (DMT, Denmark) and equilibrated in 5 ml of gassed (95% O_2_ and 5% CO_2_) Krebs-Henseleit (pH 7.4) at 37°C.

Two stainless steel wires (40 *μ*m diameter) were guided through the lumen of the first branch of mesenteric artery previously cleaned from perivascular tissue. One wire was attached to a force-measurement transducer and the other was connected to a micrometer. After an initial 30-minute stabilization, vessel internal circumference and wall tension were set [21] by using the “Normalization Module” (DMT, Denmark). After a new stabilization period at resting tension for 30 minutes, vessels were contracted with 120 mM KCl to assess the arterial viability. Thoracic aortic segments were stripped off adherent tissue and mounted on 200 *μ*m wires and were stretched until a resting tension of 2.5 mN/mm and stabilized for 1 hour.

#### 2.2.1. Experimental Protocol

Concentration-response curves to endothelium-dependent dilator acetylcholine (10 pM to 30 *μ*M), NO-donor dilator sodium nitroprusside (10 pM to 30 *μ*M), and to constrictor KCl (1 mM to 108 mM), thromboxane mimetic 9,11-dideoxy-9a,11a-methanoepoxy prostaglandin F_2*α*_ (U46619) (1 pM to 3 *μ*M), noradrenaline (10 pM to 30 *μ*M), and serotonin (1 nM to 100 *μ*M) were assessed in small mesenteric artery and thoracic aorta segments. In order to construct concentration-response curves to acetylcholine and sodium nitroprusside, vessels were precontracted with U46619 to reach 80% of the maximal response. Some vessel rings were incubated for 15 minutes with the nonselective inhibitor of NOS N*ω*-nitro-L-arginine methyl ester (L-NAME) (100 *μ*M), the selective inhibitor of inducible NOS (iNOS) N6-(1-iminoethyl)-L-lysine (L-NIL) (10 *μ*M), and the nonselective inhibitor of cyclooxygenase indomethacin (10 *μ*M) and then concentration-response curves to noradrenaline were obtained.

### 2.3. Real-Time PCR

Small mesenteric arteries and thoracic aorta cleaned from fat and connective tissue were pulverized in liquid nitrogen. Total RNA samples were isolated by the Trizol method (Invitrogen, USA) and the purity and concentration were determined by using the Nanodrop spectrophotometer (Thermo-Scientific, USA). cDNA was prepared using 2 *μ*g of total RNA from each sample and M-MLV reverse transcriptase (Promega, USA). Real-time PCR was performed using SYBR® Green PCR Master Mix (Qiagen, USA), 100 ng cDNA, and the specific primers (Table 1) at the following thermal condition: 95°C for 2 minutes, 40 cycles of 95°C for 15 seconds, and 60°C for 1 minute. Primers were constructed from information taken from GenBank. The specificity of the reaction with SYBR Green was confirmed by analysis of dissociation curve. The reactions were performed and analyzed by the Corbett Research system (Corbett Life Sciences, Australia). Cyclophilin was used as a housekeeping gene.

### 2.4. Collagen Content Determination

Small mesenteric artery and thoracic aorta segments were fixed in 4% paraformaldehyde for 2 hours and then transferred to 70% ethanol. Next, vessels were dehydrated through a consecutive series of graded ethanol baths (70, 80, 90, and 95%), diaphanized in xylene, and embedded in paraffin. Transversal sections (7 *μ*m) were deparaffinized and hydrated. Sections were then incubated with 0.1% picrosirius red solution for 30 minutes for collagen staining, following wash in acidified water (0.01 N acetic acid). Images were obtained in bright-field microscopy (Nikon, Japan) using ×40 objective (mesenteric arteries) or ×10 objective (thoracic aorta). Content of collagen was quantified at the media and adventitia layers by using ImageJ analysis software (NIH, USA).

### 2.5. Drugs

Acetylcholine, sodium nitroprusside, U46619, noradrenaline, serotonin, L-NAME, indomethacin, and Sirius Red were purchased from Sigma-Aldrich (USA). L-NIL was obtained from Cayman Chemical (USA).

### 2.6. Data Analysis

Relaxation was expressed as the percentage of reduction of U46619-induced tone. Contraction was expressed as the change in the wall tension (mN/mm). For each individual concentration-response curve, the maximal response (*E*_max_) and the logarithm of concentration inducing 50% of maximal response* (EC50)* were calculated by a nonlinear regression analysis using GraphPad Prism 5 (GraphPad Software Inc., USA). The changes in mRNA expression were calculated using the 2^−ΔΔCt^ method [Livak and Schmittgent, 2001] and were expressed in relation to data of the control group, which was assigned a value of 1. Collagen content was expressed as percentage of area stained with picrosirius red (media plus adventitia layers) in relation to the total area of artery wall. All data are presented as mean ± SEM. Statistical analysis was performed with unpaired *t*-test or one-way analysis of variance (ANOVA) followed by Tukey post hoc test for multiple comparisons. Probability values (*P*) were considered significant when less than 0.05.

## 3. Results

### 3.1. Characteristics of Obese Mice

After 16 weeks of feeding on the high-fat diet, mice presented a higher body weight gain and weight of retroperitoneal and periepididymal fat pad compared to those fed on the control diet (Table 2). Serum levels of total cholesterol, HDL cholesterol, and LDL cholesterol, but not triglycerides, were increased in obese mice (Table 2). Glycaemia and serum insulin concentration were higher in obese mice when compared to control mice (Table 2). The serum concentration of the proinflammatory cytokine TNF-*α*, but not IL-6, was elevated in obese mice (Table 2).

### 3.2. Relaxation in the Small Mesenteric Arteries and Thoracic Aorta Is Not Modified in High-Fat Diet-Induced Obese Mice

Internal diameter of first branch segments of mesenteric artery used at the vascular reactivity study was similar between control and obese groups (internal diameter—control mice: 222.9 ± 7.3 *μ*m; obese mice: 211.5 ± 10.1 *μ*m; *n* = 26).

**Figure 1:**
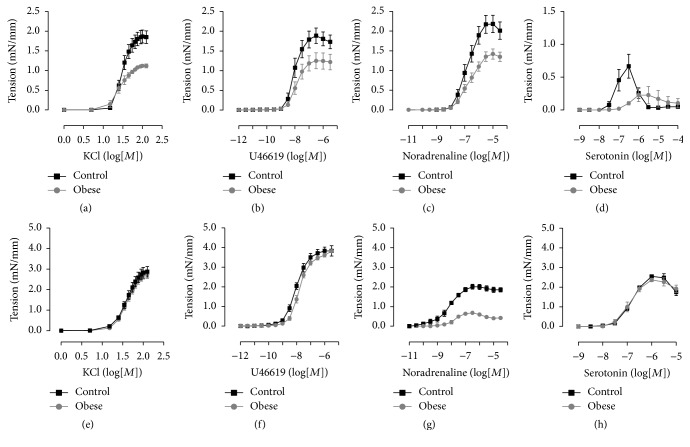
Contractile ability in arteries of obese mice. Concentration-response curves to potassium chloride (KCl), U46619, noradrenaline, and serotonin in small mesenteric arteries (a, b, c, and d) and thoracic aorta (e, f, g, and h) from control and obese mice. Data are expressed as mean ± SEM. *N* = 5 (KCl and serotonin). *N* = 9–12 (U46619 and noradrenaline).

The endothelium-dependent relaxation induced by acetylcholine and the relaxation induced by the NO-donor sodium nitroprusside in both types of arteries of obese mice were similar to control mice (Table 3). On the other hand, the maximal contraction induced by all KCl, U46619, noradrenaline, and serotonin was reduced in small mesenteric arteries obese mice when compared to control mice (Figures 1(a), 1(b), 1(c), and 1(d); Table 3). However, only the maximal contraction induced by noradrenaline and the sensitivity to U46619 were reduced in thoracic aorta of obese mice in comparison to control mice (Figures 1(e), 1(f), 1(g), and 1(h); Table 3).

### 3.3. Modulation of Noradrenaline-Induced Contraction by NO and Prostaglandins Is Increased in Thoracic Aorta, but Not in Small Mesenteric Aorta, of Obese Mice

**Figure 2:**
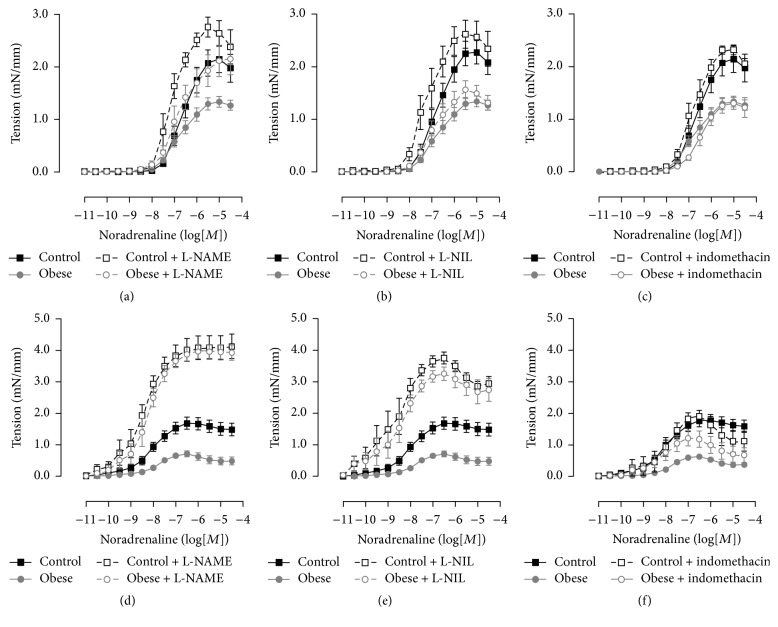
Modulation of contractile response by nitric oxide and prostanoids in obese mice. Concentration-response curves to noradrenaline in the absence and presence of inhibitors of nitric oxide synthase (L-NAME), inducible nitric oxide synthase (L-NIL), and cyclooxygenase (indomethacin) in small arteries (a, b, and c) and thoracic aorta (d, e, and f) from control and obese mice. Data are expressed as mean ± SEM. *N* = 6–12.

In small mesenteric arteries, incubation with the nonselective inhibitor of NOS L-NAME increased the sensitivity to noradrenaline in control mice and the maximal contraction induced by noradrenaline in obese mice in comparison to respective arteries nonincubated with the inhibitor (Figure 2(a); Table 4). However, the sensitivity to noradrenaline in the small mesenteric arteries incubated with L-NAME of obese mice was still reduced when compared to arteries of control mice in the same condition (Figure 2(a); Table 4). The incubation with the selective inhibitor of iNOS L-NIL and the nonselective inhibitor of cyclooxygenase indomethacin did not modify the contraction induced by noradrenaline in small mesenteric arteries from either control or obese mice (Figures 2(b) and 2(c); Table 4).

Incubation of thoracic aorta with L-NAME increased the noradrenaline-induced maximal contraction in both control and obese groups, abolishing the difference between the groups (Figure 2(d); Table 4). Surprisingly, the selective inhibitor of iNOS L-NIL promoted similar effects in relation to the nonselective inhibitor of NOS, increasing the contraction to noradrenaline in thoracic aorta of control and obese mice (Figure 2(e); Table 4). Incubation with indomethacin did not change the contraction to noradrenaline in thoracic aorta from control mice but increased the response in obese mice, leaving the contraction similar to that of control mice (Figure 2(f); Table 4).

### 3.4. mRNA Expression of Inflammatory Markers in Small Mesenteric Arteries and Thoracic Aorta

**Figure 3:**
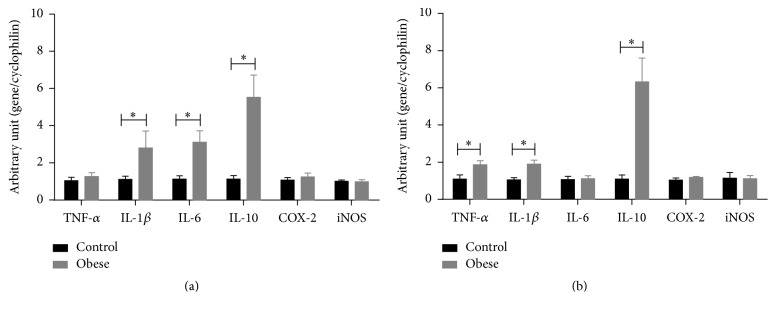
Inflammatory markers in arteries of obese mice. mRNA levels of tumor necrosis factor (TNF) *α*, interleukin- (IL-) 1*β*, IL-6, and IL-10, cyclooxygenase 2 (COX-2), and inducible nitric oxide synthase (iNOS) in small mesenteric arteries (a) and thoracic aorta (b) from control and obese mice. Expression levels of specific gene mRNA were normalized to the cyclophilin mRNA. Data are expressed as mean ± SEM. Unpaired* t*-test: ^*∗*^*P* < 0.05 versus control mice. *N* = 5–10.

Levels of mRNA expression of IL-1*β*, IL-6, and IL-10, but not of TNF-*α*, cyclooxygenase 2, and iNOS, were increased in small mesenteric arteries from obese mice when compared to control mice (Figure 3(a)). In thoracic aorta, the mRNA expression levels of TNF-*α*, IL-1*β*, and IL-10, but not of IL-6, cyclooxygenase 2, and iNOS, were increased in obese mice in comparison to control mice (Figure 3(b)).

### 3.5. mRNA Expression of Phenotype Markers of Vascular Smooth Muscle in Small Mesenteric Arteries and Thoracic Aorta

**Figure 4:**
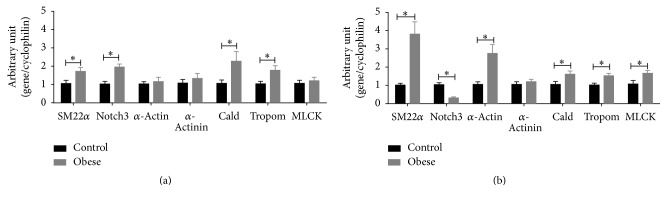
Vascular smooth muscle phenotype markers in arteries of obese mice. mRNA levels of SM22*α*, Notch3, *α*-actin, *α*-actinin, caldesmon (Cald), tropomyosin (Tropom), and myosin light chain kinase (MLCK) in small mesenteric arteries (a) and thoracic aorta (b) from control and obese mice. Expression levels of specific gene mRNA were normalized to the cyclophilin mRNA. Data are expressed as mean ± SEM. Unpaired *t*-test: ^*∗*^*P* < 0.05 versus control mice. *N* = 6–10.

In small mesenteric arteries, mRNA expression levels of SM22*α*, Notch3, caldesmon, and tropomyosin were increased, but mRNA expressions of *α*-actin, *α*-actinin, and myosin light chain kinase were similar in obese mice in relation to control mice (Figure 4(a)). On the other hand, the levels of mRNA expression of SM22*α*, *α*-actin, caldesmon, tropomyosin, and myosin light chain kinase were increased, mRNA expression of *α*-actinin was similar and mRNA expression of Notch3 was reduced in thoracic aorta from obese mice in comparison to control mice (Figure 4(b)).

### 3.6. Collagen Content Is Increased in Small Mesenteric Arteries, but Not in Thoracic Aorta, of Obese Mice

**Figure 5:**
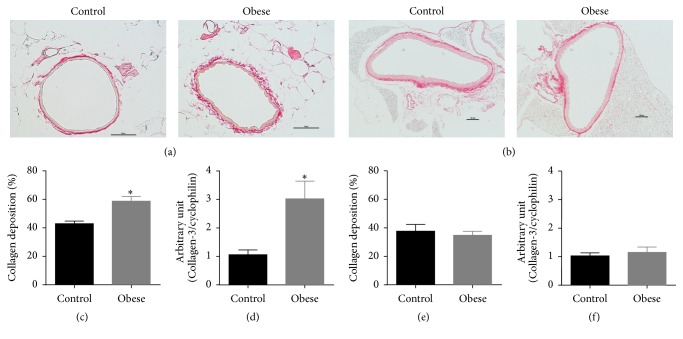
Collagen content in arteries of obese mice. Representative photomicrographs of small mesenteric artery (a) (×40 objective, scale bar represents 50 *μ*m) and thoracic aorta (b) (×10 objective, scale bar represents 100 *μ*m) sections stained with picrosirius of control and obese mice. Collagen content in the media and adventitia layer and type III collagen mRNA in small mesenteric artery (c and d) and thoracic aorta (e and f) from control and obese mice. Data are expressed as mean ± SEM. Unpaired *t*-test: ^*∗*^*P* < 0.05 versus control mice. *N* = 5.

The area stained with picrosirius red and the mRNA expression level of type III collagen were increased in small mesenteric arteries of obese mice when compared to control mice (Figures 5(a), 5(c), and 5(d)). In thoracic aorta, both collagen content and mRNA expression level of type III collagen were similar between control and obese mice (Figures 5(b), 5(e), and 5(f)).

## 4. Discussion

In the present study, we found that the endothelial function of both small mesenteric arteries and thoracic aorta was preserved in 16-week high-fat diet-induced obese mice. On the other hand, the contractile response of smooth muscle cell was compromised, but the small mesenteric arteries presented a generalized reduction of contraction to several different contractile agents, whereas only the response to noradrenaline was reduced in thoracic aorta of obese mice. In addition, small mesenteric arteries presented markers of the synthetic phenotype and thoracic aorta presented markers of the contractile phenotype of smooth muscle cells.

Endothelial dysfunction is an underlying alteration of atherosclerosis and impairs vascular function and is present in cardiovascular and metabolic diseases [9]. Despite the fact that dyslipidemia, insulin resistance, and prothrombotic and proinflammatory factors have a role in the obesity-associated vascular diseases [22] and a systemic inflammation and metabolic alterations have already been observed in 16-week high-fat diet-induced obese mice, the two types of vascular beds evaluated in the present study did not exhibit endothelial dysfunction, as observed in the relaxation elicited by acetylcholine. Moreover, our results show that the maintenance of the endothelial function in the two types of arteries is not due to an increase in the responsiveness of the smooth muscle to NO.

A local inflammation associated with oxidative stress and endothelial dysfunction was observed in thoracic aorta from female obese mice [23]. In the present study, gene expression of some inflammatory markers was increased in both small mesenteric arteries and thoracic aorta of obese mice, but we also found increase in mRNA expression level of anti-inflammatory cytokine IL-10. IL-10 deficiency during lipopolysaccharide-induced inflammation and hyperglycemia promotes reduced endothelium-dependent relaxation to acetylcholine in carotid arteries [24, 25], showing that IL-10 protects the endothelial function. Therefore, our results indicate that the compensatory increase in expression of IL-10 may be involved in the maintenance of the relaxing response in both small mesenteric arteries and thoracic aorta of obese mice.

High-fat diet-induced obesity is the animal model most used to study the effects of obesity on the cardiovascular system. However, it is important to note that the percentage of fat and the time in which animals are fed with a high-fat diet may exert different effects from those observed in the present study [26–33]. In addition, a high-fat diet that promotes a more pronounced effect in increasing the glycaemia could early impair the endothelial function [34]. Whether endothelial dysfunction occurs when IL-10 protection is missing by a longer time of feeding high-fat diet or in the presence of a more pronounced impairment of glucose metabolism is unknown and needs further investigation.

Different from the relaxing response, the contractile response was impaired in both small mesenteric arteries and thoracic aorta of obese mice. Nevertheless, our results suggest that reduced contraction in small mesenteric arteries is due to a global impairment of the smooth muscle, whereas, in thoracic aorta, it is specific to an adrenergic agonist.

Because the contractile response was evaluated in endothelium-intact arteries and the relaxing endothelial factors exert a negative modulation of the vascular contraction, which could contribute to the reduced contraction observed in obese mice, we have evaluated the noradrenaline-induced contraction in the presence of NOS and COX inhibitors. The nonselective NOS inhibition with L-NAME and COX inhibition increased the contraction induced by noradrenaline in thoracic aorta of obese mice, abolishing the differences between the groups, and the contraction was still reduced in small mesenteric arteries in the presence of the inhibitors. This indicates that only the reduced contraction in thoracic aorta could be explained by an increase in the negative modulation by NO and relaxing prostanoids.

iNOS, first identified in activated macrophages, is a mediator of inflammation in different tissues and has been involved in the obesity-associated metabolic disorders [35–37]. Moreover, iNOS was involved in the reduced contractile response of thoracic aorta from an obesity model without comorbidities [38]. In the present study, the selective iNOS inhibitor L-NIL did not modify the contraction induced by noradrenaline in small mesenteric arteries from both control and obese mice, indicating that the iNOS was not involved in the impairment of the contractile response. Curiously, the effect of L-NIL on the contractile response in thoracic aorta was similar to that promoted by L-NAME in both control and obese mice. L-NIL is considered to be a relative selective iNOS inhibitor, presenting IC50 values of 5.9, 138, and 35 *μ*M for inhibition of recombinant human iNOS, eNOS, and nNOS isoforms, respectively [39]. Our results suggest that, in thoracic aorta of C57BL/6, L-NIL may inhibit other NOS isoforms in a lower concentration, despite the fact that other studies have found an in situ selective iNOS inhibition in this artery [38, 40]. However, a role of iNOS could be excluded in the reduction of noradrenaline-contraction of thoracic aorta in obese mice because the gene expression of iNOS was similar in control and obese mice.

Vascular smooth muscle cells in the media layer present different phenotypes along with a continuum from contractile/quiescent to synthetic phenotype [10]. In healthy/normal conditions, the contractile phenotype is predominant and the vascular smooth muscle cells express a set of genes necessary for maintaining a high contractile ability [41]. Under certain environmental stimuli, the expression of those genes are downregulated and differentiated smooth muscle cells switch to a synthetic phenotype, which is characterized by a high proliferation rate and synthesis of extracellular matrix [41]. The plasticity of the vascular smooth muscle cells allows them to carry out different functions and to participate in different processes, such as repairing vascular injury and vascular remodeling [42, 43].

We observed that the expression of almost all contractile phenotype-related genes evaluated in the present study was increased in thoracic aorta of obese mice and, in contrast, the expression of only half of those genes was increased in small mesenteric arteries. These results indicate that smooth muscle cells with a more contractile phenotype were present in thoracic aorta than in small mesenteric arteries of obese mice, which explains the fact that impairment in the global contractile ability of smooth muscle was observed in small mesenteric arteries more than a specific impairment in the adrenergic response, as observed in thoracic aorta.

Notch signaling is required for normal vascular development [44]. Deficiency of Notch3, which is expressed by smooth muscle cells, does not affect the vascular response to pharmacological agents, whereas it has a central role in the control of vascular response to pressure and flow in resistance arteries but not in large arteries [45]. In addition, it was demonstrated that cocultured endothelial cell, via Notch signaling, induces differentiation and reduces proliferation at the same time that it increases collagen synthesis and secretion in smooth muscle cells [46]. In the present study, the increased Notch3 gene expression in small mesenteric arteries of obese mice could explain the increased collagen deposition without reduction of contractile phenotype gene markers, which are downregulated in a synthetic phenotype. This is reinforced by the fact that the Notch3 gene expression was reduced in thoracic aorta, which did not present alteration in the collagen deposition.

In summary, the present study demonstrated that, in 16-week high-fat diet-induced obese mice, although they present metabolic alterations, the endothelial function remains preserved, whereas the smooth muscle cells are first impaired. In addition, we showed that in small mesenteric arteries there is a global impairment of the smooth muscle, whereas in thoracic aorta there is only specific alteration in response to an adrenergic agonist, and these differences could be associated with artery-specific phenotypic changes of smooth muscle cells.

## Figures and Tables

**Table 1:** Primers used in real-time PCR analysis.

Gene	Gene name	Sequence (5′ → 3′)
THRIL	TNF-*α*	F – ATGAGCACAGAAAGCATGATC
		R – TACAGGCTTGTCACTCGAATT
IL1B	IL-1*β*	F – TGACCCATGTGAGCTGAAAG
		R – GGGATTTTGTCGTTGCTTGT
IL6	IL-6	F – ACCACCCACAACAGACCAGT
		R – CAGAATTGCCATTGCACAAC
IL10	IL-10	F – CATGGGTCTTGGGAAGAGAA
		R – GCTTTCGAGACTGGAAGTGG
PTGS2	COX-2	F – AGATCAGAAGCGAGGACCTG
		R – CCATCCTGGAAAAGTCGAAG
NOS2	iNOS	F – GGATATCTTCGGTGCGGTCTT
		R – GCTGTAACTCTTCTGGGTGTCAGA
COL3A1	Type III collagen	F – GGGATCCAATGAGGGAGAAT
		R – GGCCTTGCGTGTTTGATATT
NOTCH3	Notch3	F – GCGTTTGCCAGAGTTCAGTG
		R – GAGCAGTCTGGGCCTTGGAA
TAGLN	SM22*α*	F – TGCGTGGCTACATTGGGAAT
		R – AATGCTTGGAGCAAGGGTCA
ACTA2	SM *α*-actin	F – CCATCTTTCATTGGGATGGAGTCA
		R – AATGCCTGGGTACATGGTGGTA
ACTN1	*α*-Actinin	F – CCACTTTGACCGGGATCACT
		R – CGGTTGGGGTCTACAATGCT
CALD1	Caldesmon	F – GCCACACTCTCTCAAATTGCC
		R – CACCTGGTCTGTCACCTGTC
MYLK	MLCK	F – CCAGTTACCTGGGAAGACCAT
		R – ACCTTCGAGGGATCCACACT
TPM2	Tropomyosin	F – TATGAGGAGGTGGCCAGGAA
		R – TGGTCCATGGTTCGAAGCTC
PPIA	Cyclophilin	F – TATCTGCACTGCCAAGACTGAAT
		R – CTTCTTGCTGGTCTTGCCATTCC

TNF, tumor necrosis factor; IL, interleukin; COX, cyclooxygenase; iNOS, inducible nitric oxide synthase; SM, smooth muscle; MLCK, myosin light chain kinase; F, forward; R, reverse.

**Table 2:** Body composition and serum metabolic and inflammatory markers of C57BL/6 mice after 16 weeks of feeding with control (control mice) and high-fat diet (obese mice).

	Control mice	Obese mice
Gain of body weight (g)	9.5 ± 0.3	14.5 ± 0.6^*∗*^
Retroperitoneal fat/body weight (mg/g)	3.4 ± 0.5	13.2 ± 1.2^*∗*^
Periepididymal fat/body weight (mg/g)	10.1 ± 1.1	33.4 ± 3.6^*∗*^
Triglycerides (mg/dl)	47.1 ± 4.2	38.3 ± 5.3
Total cholesterol (mg/dl)	87.4 ± 4.5	146.0 ± 8.7^*∗*^
HDL (mg/dl)	54.7 ± 2.6	92.2 ± 4.5^*∗*^
LDL (mg/dl)	25.1 ± 2.4	50.5 ± 4.2^*∗*^
Glucose (mg/dl)	127.0 ± 2.1	178.9 ± 6.0^*∗*^
Insulin (pmol/l)	25.6 ± 3.6	196.7 ± 62.6^*∗*^
TNF-*α* (pg/ml)	26.6 ± 1.7	45.8 ± 8.4^*∗*^
IL-6 (pg/ml)	57.5 ± 2.9	46.6 ± 7.3

Data are expressed as mean ± SEM. Unpaired *t*-test: ^*∗*^*P* < 0.05 versus control mice. *N* = 15.

**Table 3:** Maximal response (mN/mm) and sensitivity (log EC50) to relaxing and contractile agents in small mesenteric arteries and thoracic aorta from control and obese mice.

	Maximal response		log EC50	
	Control mice	Obese mice	Control mice	Obese mice
Mesenteric arteries				
Acetylcholine	90.50 ± 2.26	90.91 ± 1.81	−7.00 ± 0.14	−7.12 ± 0.09
SNP	92.67 ± 2.01	89.92 ± 2.42	−8.00 ± 0.21	−8.24 ± 0.15
KCl	1.61 ± 0.12	1.03 ± 0.04^*∗*^	1.72 ± 0.05	1.46 ± 0.12
U46619	1.91 ± 0.19	1.31 ± 0.21^*∗*^	−7.98 ± 0.12	−7.97 ± 0.12
Noradrenaline	2.21 ± 0.22	1.46 ± 0.13^*∗*^	−7.81 ± 0.13	−7.71 ± 0.17
Serotonin	0.74 ± 0.12	0.26 ± 0.12^*∗*^	−7.83 ± 0.21	−7.11 ± 0.19^*∗*^

Thoracic aorta				
Acetylcholine	75.94 ± 4.51	75.56 ± 5.91	−7.38 ± 0.25	−7.70 ± 0.10
SNP	95.55 ± 1.75	96.01 ± 2.21	−7.90 ± 0.17	−7.97 ± 0.08
KCl	2.88 ± 0.26	2.74 ± 0.19	1.99 ± 0.08	2.04 ± 0.06
U46619	3.90 ± 0.17	3.68 ± 0.11	−8.01 ± 0.06	−7.79 ± 0.06^*∗*^
Noradrenaline	1.99 ± 0.13	0.52 ± 0.09^*∗*^	−8.37 ± 0.23	−8.05 ± 0.11
Serotonin	2.61 ± 0.12	2.44 ± 0.10	−7.20 ± 0.02	−7.16 ± 0.06

SNP: sodium nitroprusside. KCl: potassium chloride. Data are expressed as mean ± SEM. Unpaired *t*-test: ^*∗*^*P* < 0.05 versus control mice. *N* = 5 (KCl and serotonin). *N* = 9–12 (acetylcholine, SNP, U46619, and noradrenaline).

**Table 4:** Maximal response (mN/mm) and sensitivity (log EC50) to noradrenaline in small mesenteric arteries and thoracic aorta incubated with L-NAME, L-NIL, and indomethacin.

	Maximal response		log EC50	
	Control mice	Obese mice	Control mice	Obese mice
Mesenteric arteries				
Noradrenaline	2.17 ± 0.26	1.36 ± 0.10^*∗*^	−7.63 ± 0.12	−7.77 ± 0.17
+L-NAME	2.65 ± 0.21	2.36 ± 0.36^#^	−8.29 ± 0.10^#^	−7.83 ± 0.14^*∗*^
+L-NIL	2.54 ± 0.28	1.49 ± 0.16^*∗*^	−8.23 ± 0.15	−7.97 ± 0.17
+Indomethacin	2.32 ± 0.09	1.38 ± 0.12^*∗*^	−7.82 ± 0.18	−7.42 ± 0.18

Thoracic aorta				
Noradrenaline	1.69 ± 0.19	0.49 ± 0.06^*∗*^	−8.23 ± 0.19	−8.16 ± 0.11
+L-NAME	4.05 ± 0.39^#^	3.93 ± 0.23^#^	−8.62 ± 0.25	−8.29 ± 0.18
+L-NIL	3.30 ± 0.16^#^	2.95 ± 0.28^#^	−8.98 ± 0.32	−8.76 ± 0.29
+Indomethacin	1.89 ± 0.19	1.23 ± 0.26^#^	−8.38 ± 0.22	−8.49 ± 0.11

Data are expressed as mean ± SEM. One-way ANOVA: ^*∗*^*P* < 0.05 versus control mice; ^#^*P* < 0.05 versus noradrenaline. *N* = 6–12.
